# Generalized statistics: Applications to data inverse problems with outlier-resistance

**DOI:** 10.1371/journal.pone.0282578

**Published:** 2023-03-30

**Authors:** Gustavo Z. dos Santos Lima, João V. T. de Lima, João M. de Araújo, Gilberto Corso, Sérgio Luiz E. F. da Silva

**Affiliations:** 1 School of Science and Technology, Federal University of Rio Grande do Norte, Natal, RN, Brazil; 2 Department of Theoretical and Experimental Physics, Federal University of Rio Grande do Norte, Natal, RN, Brazil; 3 Department of Biophysics and Pharmacology, Federal University of Rio Grande do Norte, Natal, RN, Brazil; 4 Department of Applied Science and Technology, Politecnico di Torino, Turin, TO, Italy; 5 GISIS, Fluminense Federal University, Niterói, RJ, Brazil; Beijing University of Posts and Telecommunications, CHINA

## Abstract

The conventional approach to data-driven inversion framework is based on Gaussian statistics that presents serious difficulties, especially in the presence of outliers in the measurements. In this work, we present maximum likelihood estimators associated with generalized Gaussian distributions in the context of Rényi, Tsallis and Kaniadakis statistics. In this regard, we analytically analyze the outlier-resistance of each proposal through the so-called influence function. In this way, we formulate inverse problems by constructing objective functions linked to the maximum likelihood estimators. To demonstrate the robustness of the generalized methodologies, we consider an important geophysical inverse problem with high noisy data with spikes. The results reveal that the best data inversion performance occurs when the entropic index from each generalized statistic is associated with objective functions proportional to the inverse of the error amplitude. We argue that in such a limit the three approaches are resistant to outliers and are also equivalent, which suggests a lower computational cost for the inversion process due to the reduction of numerical simulations to be performed and the fast convergence of the optimization process.

## 1 Introduction

The estimation of physical model parameters from observed data is a frequent problem in many areas, such as in machine learning [1, 2], geophysics [3, 4], biology [5, 6], physics [7, 8], among others [9–11]. Such a task is solved through the so-called inverse problem, which consists of identifying physical parameters that can not be directly measured from the observations [12]. Such is a process where one can use stable or unstable distributions. In the case of *α*-stable distributions, the focus of the inverse problems is on finding efficient estimators of a *α*-stable distribution that represents the data set, since the joint distribution has a density function similar to the marginal one [13, 14]. In contrast, unstable distributions have been used in inverse problems to determine efficient estimators from observed data by assuming that the parameters of the statistical distribution are known and included in the inverse problems as constraints. From a practical viewpoint, in the inverse problem, physical model parameters are estimated by matching the estimates to the observed data by optimizing an objective function [15]. The objective function in the least-squares sense is widely used, which is based on the assumption that errors are independent and identically distributed (*iid*) according to a standard Gaussian probability distribution [12]. Although this approach is quite popular, the least-squares-based estimator is biased if the errors are non-Gaussian, violating the Gauss-Markov theorem [16, 17]. Indeed, just a few outliers are enough for the least-squares criterion to be inappropriate [18].

In this way, a lot of non-Gaussian criteria have been proposed to mitigate the inverse problem sensitivity to aberrant measurements (outliers). The most common criterion to deal with non-Gaussian errors is based on the *L*_1_-norm of the difference between the estimated and the observed data, in which the errors are assumed to be *iid* according to a double exponential distribution (Laplace distribution) [19]. Although this approach is known for being outlier-insensitive, this criterion is unique in the case where the difference between the estimates and the observed data is zero (or very close to zero). Thus, it is necessary to assume that the absolute error is greater than zero according to the machine precision used, which generates an indeterminacy problem from a computational point of view. To avoid the singularity of this approach, hybrid criteria which combine the least-squares distance with the least-absolute-values criterion (*L*_1_-norm) have been proposed and successfully applied for parameter robust estimation [20–22]. However, hybrid approaches require the determination of a threshold parameter, which demands boring trial-and-error investigations, increasing the computational cost [23].

Indeed, objective functions based on heavy-tailed probability distributions, such as the Cauchy-Lorentz distribution [24] and the Student’s *t*-distribution [25], have demonstrated robust properties for unbiased data inversion. However, both approaches assume a fixed probability distribution of errors, not adapting to the particularities of the model or data at hand. In this sense, objective functions based on generalized distributions are interesting because they might be adapted to the specificity of the erratic data by selecting an adequate free-parameter. In fact, several generalized approaches have been proposed to deal with erratic data [26–30]. Thus, generalized distributions based on the Rényi, Tsallis and Kaniadakis statistics have generated objective functions robust to erratic noise [31].

**Fig 1:**
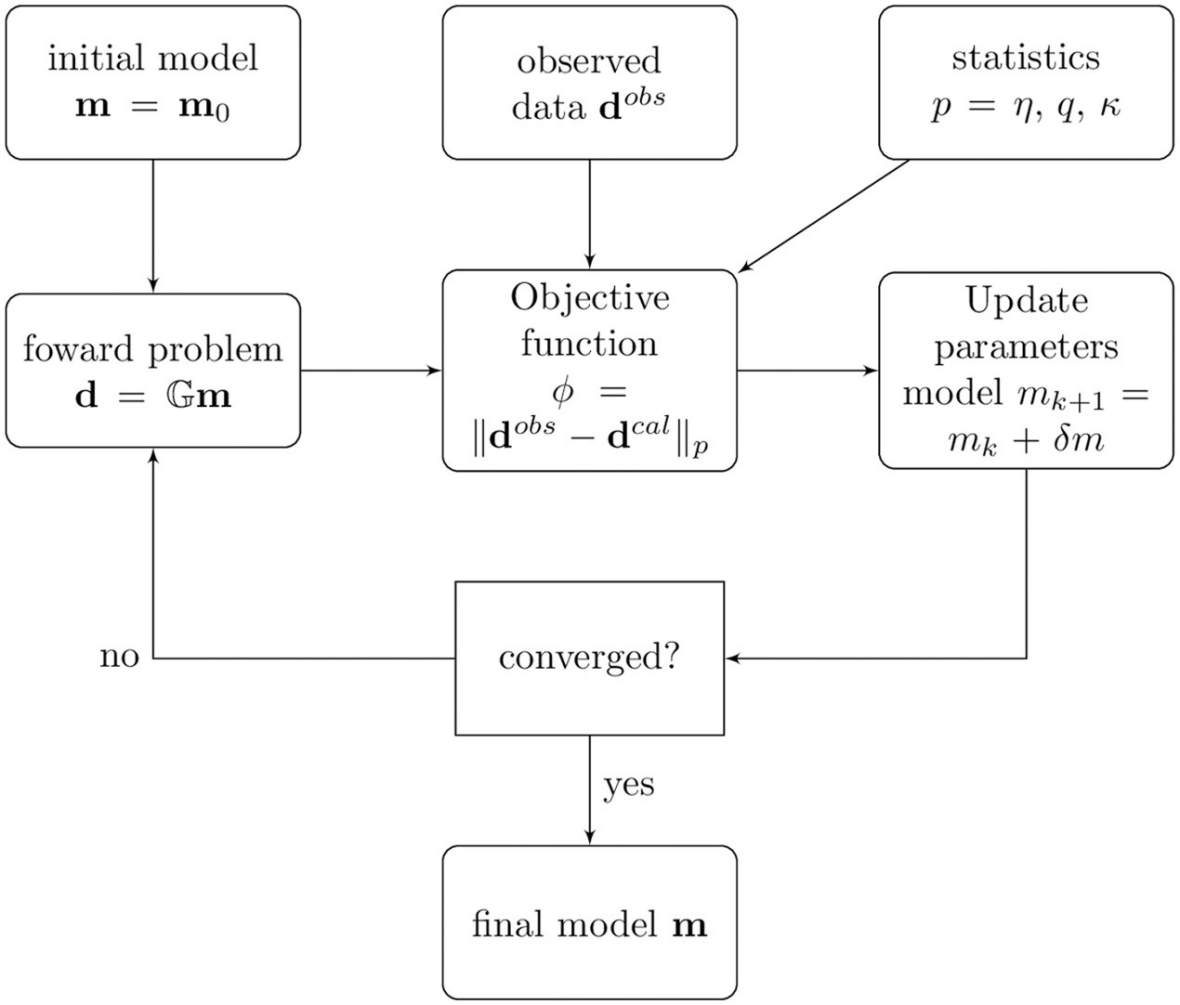
Workflow of the computational experiments.

In this work, we present a review of robust approaches based on the field of statistical physics to estimates physical parameters from a data set polluted by outliers. In particular, we consider deformed Gaussian distributions associated to generalized statistical mechanics in the sense of Rényi (*η*-statistics) [32], Tsallis (*q*-statistics) [33] and Kaniadakis (*κ*-statistics) [34]. In this context, we place objective functions based on *η*-, *q*- and *κ*-generalizations in the broad context of the Gauss’ law of error [35–37], see Refs. [31, 38, 39]. The three deformed Gaussian distributions mentioned above have already demonstrated robust properties in many applications [31, 38–44]. However, the entropic index associated with each of these approaches that make the inversion process more robust requires thorough investigation. Furthermore, we analyze and compare the generalized objective functions from a statistical and numerical point of view in order to obtain the optimum value of the entropic index. In Fig 1 we summarize the workflow of the experiments employed in this work, which is a flowchart represented of an inverse problem. We call attention that in generalized frameworks, generalized statistics define the norm employed in the inversion problem solution.

We have organized this article as follows. In Section 2 we present a brief review on the solution of inverse problems using the maximum likelihood method in the conventional framework, as well as in the framework of Rényi, Tsallis and Kaniadakis. Moreover, in Section 3.1 we discuss the similarities among the generalized objective functions by considering a numerical test whose purpose is to estimate the parameters of a line that represents the observations; and finally, Section 3 is destined to apply the methodology presented in the article to address a classic geophysical problem that consists of estimating the acoustic impedance model using seismic data post-stack contaminated with spike noise.

## 2 A brief review of generalized statistical in inverse theory: Maximum likelihood methods

An inverse problem is formulated as an optimization task, in which an objective function describes how well the estimated model **m** generates a modeled dataset (or estimates) that matches the measurements [12]. In this regard, the estimates **d**^*est*^ are obtained by the following operation [12, 15]:

$$\begin{array}{c} d^{est}=G\left(m\right), \end{array}$$ (1)

where G represents the forward operator. In the linear case, the latter equation is usually represented by the direct relationship $d^{est}=Gm$. The forward operator makes the connection between the model space and the measurements (data) space through an adequate physical law. To illustrate the nature of a forward operator, let consider the mass distribution (model **m**) of the surface of a solid plate from the measures of the mass at different plate locations (observed data **d**^*obs*^) recorded by gravimeters. In this case, the forward operator G may be represented by the Newton’s law of universal gravitation. So, the inverse problem consists of obtaining a model **m** by matching estimates **d**^*est*^ computed by Eq (1) with observed data **d**^*obs*^ by means of an objective function, as we will discuss further.

In the conventional approach, model parameters are estimated by maximizing the objective function is derived from the maximization of the Boltzmann-Gibbs-Shannon entropy (BGS):

$$\begin{array}{c} S_{BGS}[p]=-\sum_{i=1}^{N}p\left(x_{i}\right)\;\text{ln}(p(x_{i}))\;, \end{array}$$ (2)

subject to the following constraints:

$$\begin{array}{c} \sum_{i=1}^{N}p\left(x_{i}\right)=1\;\left(\text{normalization}\;\text{condition}\right), \end{array}$$ (3)

$$\begin{array}{c} \sum_{i=1}^{N}x_{i}^{2}\;p{(}_{i}x)=1\;\left(\text{unity}\;\text{variance}\right), \end{array}$$ (4)

where *p* is a probability function and **x** = **d**^*obs*^ − **d**^*est*^ = {*x*_1_, *x*_2_, …, *x*_*N*_} represents the difference between observed and estimates. As well known in the literature, the probability distribution determined from the optimization of the BGS functional entropy subject to the constraints in Eqs (3) and (4) corresponds to the standard Gaussian distribution (see, for instance, Section 2 of Ref. [42]):

$$\begin{array}{c} p\left(x\right)=\frac{1}{\sqrt{2\pi }}\text{exp}(-\frac{1}{2}x^{2}). \end{array}$$ (5)

In other words, inverse problems, in the conventional framework, are solved based on the premise that errors are independent and identically distributed (iid) according to a standard Gaussian likelihood function, which is formulated as the following optimization problem:

$$\begin{array}{c} \begin{array}{cc} \underset{m}{\text{max}}\;L_{G}\left(m\right) & =\prod_{i=1}^{N}p(x_{i}\left(m\right))\\ & ={\left(\frac{1}{\sqrt{2\pi }}\right)}^{N}\text{exp}(-\frac{1}{2}\sum_{i=1}^{N}x_{i}^{2}\left(m\right)). \end{array} \end{array}$$ (6)

A likelihood function is a useful tool to estimate physical parameters from the empirical data. In practice, inverse problems based on the Gauss’ law of error are formulated in a least-squares sense. To see this, we notice that maximizing the likelihood function in (6) is equivalent to minimizing the negative of the log-likelihood:

$$\begin{array}{c} \begin{array}{cc} \underset{m}{\text{min}}\;\phi \left(m\right) & =\frac{1}{2}\sum_{i=1}^{N}x_{i}^{2}\left(m\right)\\ & =\frac{1}{2}\sum_{i=1}^{N}{\left(d_{i}^{obs}-d_{i}^{est}\left(m\right)\right)}^{2}. \end{array} \end{array}$$ (7)

However, it is worth emphasizing that in several problems the errors are non-Gaussian and, therefore, in such contexts the conventional approach becomes inefficient, especially in the presence of aberrant measures (outliers) [45].

For an objective function to admit a minimum, it must satisfy the condition

$$\begin{array}{c} \sum_{i=1}^{N}c_{i}I\left(x_{i}\right)=0,\;\text{with}\;I\left(x\right)≔\frac{\partial \;\phi (x;m_{k})}{\partial m_{k}}, \end{array}$$ (8)

where *m*_*k*_ is the k-th element of the parameter model vector **m**.

In Eq (8) *c*_*i*_ are arbitrary constants and I is the so-called influence function [42, 46, 47]. The influence function Eq (8) informs us about the sensitivity of the objective function to outliers. In this sense, if I→±∞ for a certain observed data $d_{i}^{obs}\to \infty$, the objective function is sensitive to outliers and, therefore, it is not a robust estimator. A robust estimate, however, will indicate I→0 for $d_{i}^{obs}\to \infty$. What we have in the conventional approach, however, is that the conventional objective function, Eq (7), is linearly influenced by outliers, as in the following equation

$$\begin{array}{c} I\left(m_{k}\right)=-\sum_{i=1}^{N}(\frac{\partial d_{i}^{est}}{\partial m_{k}})(d_{i}^{obs}-d_{i}^{est}\left(m\right)). \end{array}$$ (9)

To simplify the notation we restrict ourselves to the linear case: $d^{est}\left(m\right)=Gm$. In this case, the classical influence function is given by:

$$\begin{array}{c} I\left(m_{k}\right)=-\sum_{i=1}^{N}G_{i}(d_{i}^{obs}-G_{i}m_{k}). \end{array}$$ (10)

Note that when there are outliers (*d*^*obs*^ → ∞), the classical influence function tends to infinity (I→-∞), which confirms the non-robustness of the classical framework.

The conventional theory of inverse problems, based on Gaussian statistics, fails with errors outside the Gaussian domain. In this sense, we look for alternatives to generalize Gaussian statistics in order to find more robust methods to deal with outliers.

### 2.1 Rényi’s Framework

Based on information theory, A. Rényi [32, 48] introduced a general information entropy as a one-parameter generalization of the BGS entropy. Rényi entropy (*η*-entropy) functional is expressed by:

$$\begin{array}{c} S_{\eta }(p)=\frac{1}{1-\eta }\text{ln}(\sum_{i=1}^{N}p^{\eta }\left(x_{i}\right)),\;\eta \geq 0, \end{array}$$ (11)

where *η* is the entropic index. Furthermore, the entropy in Eq (11) shares many of the properties of the BGS entropy Eq (2), such as: it is non-negative, it is additive, and it has an extreme for the case of equiprobability [32]. Rényi’s entropy recovers Shannon’s entropy at the limit *η* → 1. Applications of Rényi entropy can be found in several fields [49–51].

**Fig 2:**
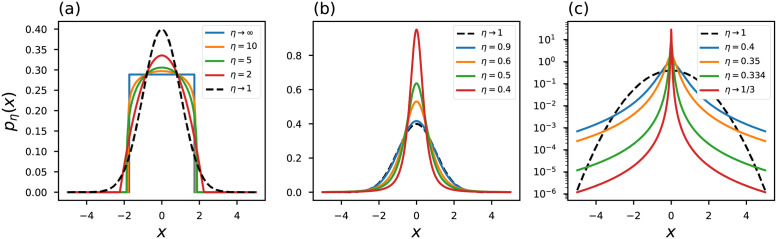
*η*-Gaussian probability distribution. The black dashed line represents the conventional curves.

Taking into account the constraints in Eqs (3) and (4), *η*-entropy is maximized by the *η*-generalized Gaussian distribution, *η*-Gaussian, which is expressed in the form [35, 52, 53]:

$$\begin{array}{c} p_{\eta }\left(x\right)=A_{\eta }{\left(1-\frac{\eta -1}{3\eta -1}x^{2}\right)}_{+}^{\frac{1}{\eta -1}} \end{array}$$ (12)

where [*x*]_+_ = 0 if *x* < 0 and [*x*]_+_ = *x*. In addition, *A*_*η*_ is the normalizing constant:

$$\begin{array}{c} A_{\eta }=\{\begin{array}{c} \sqrt{\frac{1-\eta }{[3\eta -1]\pi }}\Gamma (\frac{1}{1-\eta })/\Gamma (\frac{1+\eta }{2[1-\eta ]}),\;\;\frac{1}{3}<\eta <1\\ \sqrt{\frac{1-\eta }{[3\eta -1]\pi }}\Gamma (\frac{3\eta -1}{2[1-\eta ]})/\Gamma (\frac{\eta }{\eta -1}),\;\;\eta >1 \end{array} \end{array}$$ (13)

with *Γ*(⋅) representing the gamma function. At the limit *η* → 1, the ordinary Gaussian probability distribution Eq (5) is recovered. Fig 2 shows some curves of the *η*-Gaussian probability distribution. In particular, we note that at the limit *η* → 1/3 the probability distribution approaches a strongly peaked function.

Following the same path discussed of the previous Section, that is, using the maximum likelihood method, we find a generalized objective function [31]:

$$\begin{array}{c} \begin{array}{cc} \phi_{\eta }\left(m\right) & =\frac{1}{1-\eta }\sum_{i=1}^{N}\text{ln}{\left(1-\frac{\eta -1}{3\eta -1}x_{i}^{2}\left(m\right)\right)}_{+}\\ & =∥x_{i}{∥}_{\eta }^{2}. \end{array} \end{array}$$ (14)

This function will be called *η*-objective function and at limit *η* → 1 the conventional objective function, Eq (7), is recovered. To investigate the behavior of the *η*-objective function regarding outliers we compute the influence function, as defined in Eq (8), named *η*-influence function:

$$\begin{array}{c} I_{\eta }\left(m\right)=\sum_{i=1}^{N}\frac{2x_{i}\left(m\right)}{{\left(3\eta -1-\left(\eta -1\right)x_{i}^{2}\left(m\right)\right)}_{+}} \end{array}$$ (15)

**Fig 3:**
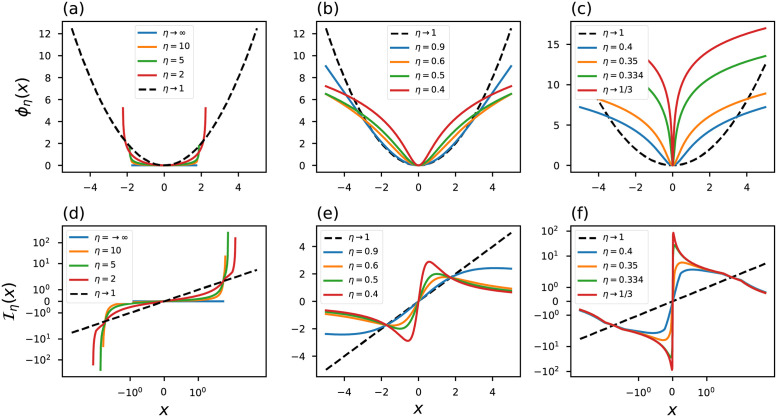
(a)-(c) objective functions and (d)-(f) influence functions generalized based on Rényi statistic. The black dashed line represents the conventional curves.

A couple of illustrative curves of the influence function are shown in Fig 3, we draw our attention to the limit case *η* → 1/3. In this region the influence of the outliers is minimized: $I_{\eta }\left(x_{i}\to \pm \infty \right)=0$, in contrast, the conventional objective function Eq (7) is strongly influenced by outliers since $I(x_{i}\to \pm \infty )=\pm \infty$.

### 2.2 Tsallis’s framework

Based on multifractals quantities and long-range interactions, C. Tsallis postulates an alternative form for the entropy to generalize the standard statistical mechanics [33]. Since then, a wide variety of applications have been performed based on Tsallis *q*-statistics [54–59]. The Tsallis approach is based on the *q*-entropy, defined as follows:

$$\begin{array}{c} S_{q}(p)=\frac{1}{q-1}(1-\sum_{i=1}^{N}p^{q}\left(x_{i}\right)), \end{array}$$ (16)

where q∈R is the entropic index (also known as nonextensive parameter). The choice of the entropic index *q* assigns new properties to the entropy functional, and in the limit case *q* → 1 it recovers the conventional BGS entropy.

By considering the maximum entropy principle for *q*-entropy, a *q*-generalization of Gauss’ law of error was formulated in Ref. [36] assuming that the errors *x* follow a probability distribution. In this regard, the probability function is computed by maximizing the *q*-entropy constrained to the normalization condition, Eq (3), and the *q*-variance [60, 61] given by:

$$\begin{array}{c} {\left\langle x\right\rangle }_{q}^{2}=\sum_{i=1}^{N}x_{i}^{2}P_{q}\left(x_{i}\right),\;\;\text{with}\;P_{q}\left(x_{j}\right)=\frac{p^{q}\left(x_{j}\right)}{\sum_{i=1}^{N}p^{q}\left(x_{i}\right)}, \end{array}$$ (17)

in which *P*_*q*_ is the escort probability function [61–63]. The probability distribution resulting from the aforementioned optimization problem is known by the *q*-Gaussian distribution:

$$\begin{array}{c} p_{q}\left(x\right)=A_{q}{\left(1+\frac{q-1}{3-q}x^{2}\right)}_{+}^{\frac{1}{1-q}} \end{array}$$ (18)

where the normalization constant is given by [64]:

$$\begin{array}{c} A_{q}=\{\begin{array}{c} \sqrt{\frac{1-q}{[3-q]\pi }}\Gamma (\frac{5-3q}{2[1-q]})/\Gamma (\frac{2-q}{1-q}),\;\;-\infty <q<1\\ \sqrt{\frac{q-1}{[3-q]\pi }}\Gamma (\frac{1}{q-1})/\Gamma (\frac{3-q}{2[q-1]}),\;\;1<q<3 \end{array} \end{array}$$ (19)

**Fig 4:**
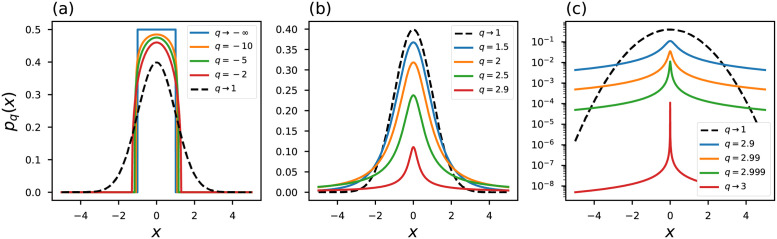
*q*-Gaussian probability distribution. The black dashed line represents the conventional curves.

A comparison between the conventional Eq (2) and Tsallis approach Eq (16) reveals that most probable events gain greater weight in the entropy calculation for the case in which *q* ≠ 1. In this sense, the usual average is replaced by an average that depends on the choice of the index and so the higher the value of this index [65], the most likely events receive higher weights. Fig 4 show illustrative curves of the *q*-Gaussian probability distribution. It is important to note that at the limit *q* → 3 we have a behavior that reminds us of the Rényi distribution in *η* → 1/3: at this limit both distributions display a peaked behavior.

Applying the probabilistic maximum-likelihood method in the *q*-Gaussian distribution, we have the following objective function [38]

$$\begin{array}{c} \begin{array}{cc} \phi_{q}\left(m\right) & =\frac{1}{q-1}\sum_{i=1}^{N}\text{ln}{\left(1+\frac{q-1}{3-q}x_{i}^{2}\left(m\right)\right)}_{+}\\ & =∥x_{i}{∥}_{q}^{2} \end{array} \end{array}$$ (20)

In order to check the influence of outliers for our objective function, we calculate the influence function [42]:

$$\begin{array}{c} I_{q}\left(m\right)=\sum_{i=1}^{N}\frac{2x_{i}\left(m\right)}{{\left(3-q+\left(q-1\right)x_{i}^{2}\left(m\right)\right)}_{+}} \end{array}$$ (21)

**Fig 5:**
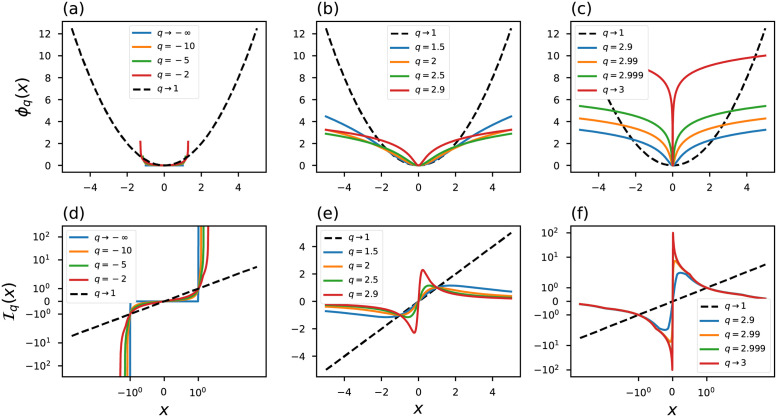
(a)-(c) objective functions and (d)-(f) generalized influence functions based on Tsallis statistic. The black dashed line represents the conventional curves.

Eq (21) reveals that the Tsallis framework also shows a robust objective function that is resistant to outliers. Fig 5 displays a couple of influence function curves. At the limit *x*_*i*_ → ±∞, the choice of index *q* < 1 implies ${\left(3-q+\left(q-1\right)x_{i}^{2}\right)}_{+}^{-1}\to \infty$ because the sum inside the brackets will always result in a negative quantity: $I_{q<1}\left(\pm \infty \right)\to \infty$. On the other hand, for *q* > 1 the sum inside the brackets turns into a large positive number, leading to $I_{q>1}\left(\pm \infty \right)\to 0$.

### 2.3 Kaniadakis’s framework

G. Kaniadakis proposed a new way to calculate the entropy based on the principle of Kinetic Interaction [34, 66]. This new *κ*-entropy that generalizes the BGS statistics is given by:

$$\begin{array}{c} S_{\kappa }(p)=-\frac{1}{2\kappa }\sum_{i=1}^{N}(p^{1+\kappa }\left(x_{i}\right)-p^{1-\kappa }\left(x_{i}\right)) \end{array}$$ (22)

Kaniadakis statistics has been applied in different contexts [67–69]. The conventional entropy (BGS) is recovered in the limit of the entropic index *κ* → 0. Kaniadakis’ framework not only includes conventional BGS statistics, but it is related to other statistics, such as the famous quantum statistics of Fermi-Dirac and Bose-Einstein, as well as the Tsallis [34, 70].

Based on the *κ*-Gaussian statistic, the references [71, 72] presents an error law that can be applied to a variety of problems and that, because it has a heavy tails distribution, it is able to satisfactorily work with outliers. The *κ*-Gaussian distribution is given by

$$\begin{array}{c} p_{\kappa }\left(x\right)=\frac{1}{A_{\kappa }}{\left(\sqrt{1+\kappa^{2}\beta_{\kappa }^{2}x^{4}}-\kappa \beta_{\kappa }x^{2}\right)}^{1/\kappa } \end{array}$$ (23)

where *A*_*κ*_ is the normalization constant given by

$$\begin{array}{c} A_{\kappa }=(1+\frac{|\kappa |}{2})\sqrt{\frac{2|\kappa |\beta_{\kappa }}{\pi }}\frac{\Gamma (1/|2\kappa |+1/4)}{\Gamma (1/|2\kappa |-1/4)} \end{array}$$ (24)

and *β*_*κ*_ > 0 depends on the *κ* index and is given by:

$$\begin{array}{c} \begin{array}{cc} \beta_{\kappa } & =\frac{1+|\kappa |/2}{|2\kappa |(2+3|\kappa |)}\\ & \times \frac{\Gamma (1/|2\kappa |-3/4)}{\Gamma (1/|2\kappa |+1/4)}\frac{\Gamma (1/|2\kappa |+3/4)}{\Gamma (1/|2\kappa |-1/4)} \end{array} \end{array}$$ (25)

**Fig 6:**
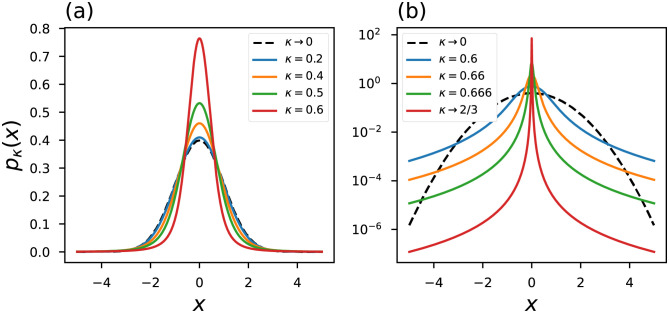
*κ*-Gaussian probability distribution. The black dashed line represents the conventional curves.

Some curves of the *κ*-Gaussian distribution are shown in Fig 6. We notice that the distribution has heavy tails when choosing *κ* → 2/3 and at this limit, as well as in the *η*-Gaussian and *q*-Gaussian distributions, the distribution shows a peaked behavior that resembles the Dirac delta distribution.

In this scenario, the inverse problem is therefore formulated as the problem of optimizing the *κ*-objective function that derives from the principle of maximum likelihood

$$\begin{array}{c} \begin{array}{cc} \phi_{\kappa }\left(m\right) & =-\frac{1}{\kappa }\sum_{i=1}^{N}\text{ln}(\sqrt{1+\kappa^{2}\beta_{\kappa }^{2}x_{i}^{4}\left(m\right)}-\kappa \beta_{\kappa }x_{i}^{2}\left(m\right))\\ & =∥x_{i}{∥}_{\kappa }^{2} \end{array} \end{array}$$ (26)

and the analysis of the robustness of this objective function can be performed by the *κ*-influence function, which is given by:

$$\begin{array}{c} I_{\kappa }\left(m\right)=\sum_{i=1}^{N}\frac{2\beta_{\kappa }x_{i}\left(m\right)}{\sqrt{1+\kappa^{2}\beta_{\kappa }^{2}x_{i}^{4}\left(m\right)}} \end{array}$$ (27)

**Fig 7:**
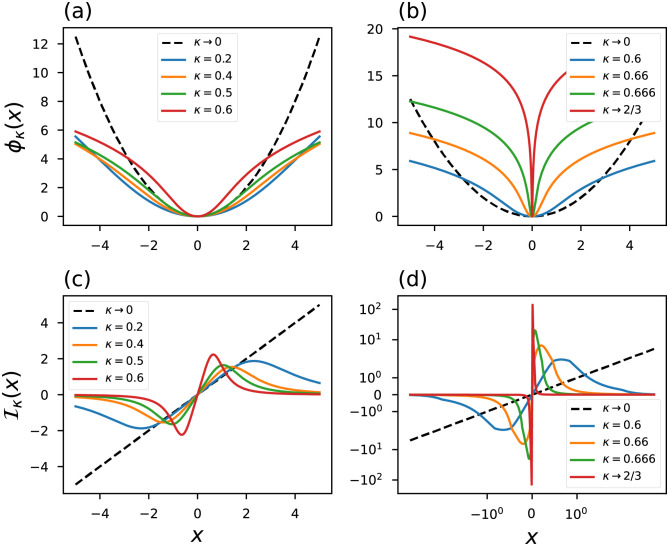
(a)-(b) objective functions and (c)-(d) generalized influence functions based on Kaniadakis statistic. The black dashed line represents the conventional curves.

The curves of the *κ*-objective and *κ*-influence functions are shown in Fig 7. In Fig 7(c) and 7(d) we notice that as we increase the index *κ* the values of the influence function far from *x* = 0 vicinity are negligible. In particular, at the limit *κ* → 2/3 we observe the curve that indicates less influence for *x* → ±∞ errors. Looking at Eq (27) we noticed that for *x*_*i*_ → ±∞ we have $I_{\kappa }\left(x_{i}\to \pm \infty \right)=0$.

## 3 Numerical experiments

### 3.1 Comparing the performance of objective functions

In this section, we present a simple numerical experiment in order to quantitatively analyze the robustness of objective functions based on generalized statistics. The experiment consists of estimating the coefficients **m** = {*m*_1_, *m*_2_} of a linear polynomial, *d*^*est*^ = *m*_1_*x* + *m*_2_, from observed data **d**^*obs*^ contaminated by outliers. In this regard, we consider the independent variable $x\in R^{50}$ within the range [−1, 1] to generate 50 numbers obeying a linear polynomial with coefficients *m*_1_ = 1 and *m*_2_ = 2. Then we contaminate the numbers generated with a Gaussian distribution with zero-mean and variance *σ*^2^ = 0.2. In addition, the variable *d*^*obs*^ is contaminated with outliers in the region 0.4 ≤ *x* < 0.9, of *d*^*obs*^, the outliers are given by $d_{i}^{obs}=10f$ where *f* is a Gaussian random variable with zero-mean and standard-deviation *σ*^2^ = 1.

**Fig 8:**
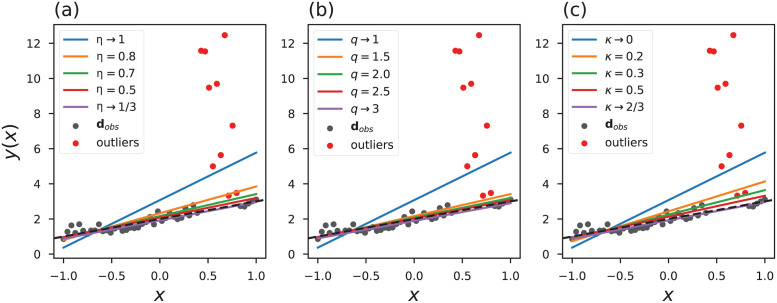
Fit of the estimated lines using the objective functions of (a) Rényi, (b) Tsallis and (c) Kaniadakis. The dashed line indicates an ideal line, and the points highlighted in red indicate the inserted outliers.

Conventionally, this problem is treated by minimizing the square of the residuals based on Gaussian statistics [15, 73]. However, the Gaussian estimate is not appropriate to solve problems with discrepant values. In this sense, we propose to estimate the coefficients **m** using the objective functions of Rényi (Eq (14)), Tsallis (Eq (20)) and Kaniadakis (Eq (26)). The values of the entropic index were used between 0.3334 ≤ *η* ≤ 1, 1 ≤ *q* ≤ 2.9999 and 0 ≤ *κ* ≤ 0.6666. Inside each interval, 200 uniform spaced values were taken. Fig 8 shows the fit between the observed data and the estimated lines with generalized objective functions.

**Fig 9:**
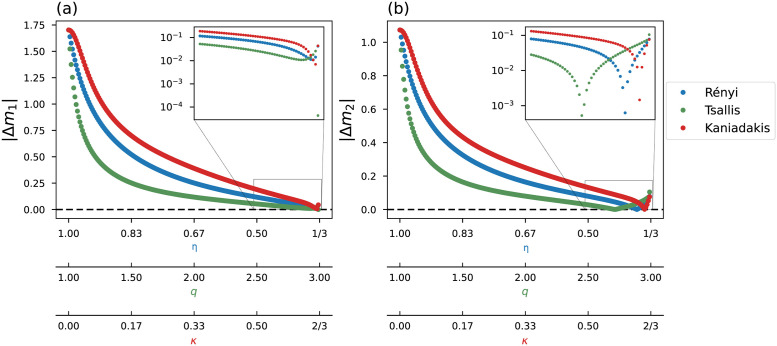
Relation between estimated parameters and entropic indexes. The zoom in window in (a) and (b) emphasizes the regions 0.5 ≤ *η* < 1/3, 2.5 ≤ *q* < 3 and 0.5 ≤ *κ* < 2/3.

The estimated models, $m^{est}=\left\{m_{1}^{est},\;m_{2}^{est}\right\}$, are compared with the ideal model **m** = {1, 2} to find for each objective function the best entropic index that achieve an optimal fitting. Fig 9 correlates the estimated values of the intercept, $\Delta m_{1}=m_{1}^{est}-1$, and the slope, $\Delta m_{2}=m_{2}^{est}-2$, with the employed entropic index. We can see that as we move away from the Gaussian limit, we find a better fit.

We compared the lines obtained with the estimated parameters, calculating the Mean Absolute Error, *MAE* = ∑_*i*_∣**m**^*est*^ − **m**∣/*N* and the symmetric Mean Absolute Percentage Error, sMAPE = 2/*N*∑_*i*_∣**m**^*est*^ − **m**∣/(∣**m**^*est*^∣ + ∣**m**∣). The obtained results are: *MAE*_*η*_ = 0.0136 with index *η* = 0.3635 and *sMAPE*_*η*_ = 0.0861 with index *η* = 0.3602; *MAE*_*q*_ = 0.0134 with *q* = 2.7587 and *sMAPE*_*q*_ = 0.0853 with index *q* = 2.7487; and *MAE*_*κ*_ = 0.0127 with *κ* = 0.6532 and *sMAPE*_*κ*_ = 0.0806 with index *κ* = 0.6566. The *MAE* represents the average deviation between predicted and reference values, and the best result is achieved for *MAE* close to zero. In contrast, the *MAE* calculated with the conventional objective function was 1.2000, while the *sMAPE* was 0.5791.

With this test, we observe that the results improve as we move away from the Gaussian limit. In particular, we notice that in Fig 9 the green curve corresponding to the Tsallis result shows the steepest decrease after the Gaussian limit (*q* = 1), while the red curve (Kaniadakis) presents a curve that slowly decreases after the Gaussian limit.

An analysis of the influence functions, Eqs (15), (21) and (27), reveals that they are zero for *x*_*i*_ → ±∞, regardless of the choice of entropic indexes (obviously, disregarding the conventional limit). In addition, we notice that in the limits *η* → 1/3, *q* → 3 and *κ* → 2/3 the influence functions are merely a function that depends on the inverse of *x*_*i*_. Thus, the three objective functions, Eqs (14), (20) and (26), are resistant to outliers and have an entropic index limit in which they are equivalent.

### 3.2 Post-Stack inversion

In this section, we present numerical experiments to demonstrate the outlier-resistance of the data-inversion method based on generalized statistics by considering an important problem that comes from geophysics, which is an important process to obtain estimates of subsurface properties. In particular, we address a problem of seismic inversion known as Post-Stack Inversion (PSI) [74]. The goal of PSI is to estimate, from the observation of the seismic data, the acoustic impedance, which is a property of the rock defined as the product of the density of the rock and the speed of the acoustic wave in the subsurface [75].

The forward problem is formulated through the following convolutional model relationship [75]:

$$\begin{array}{c} d_{j}^{est}\left(t\right)=\frac{1}{2}\int_{-\infty }^{\infty }w\left(\tau \right)\frac{d}{dt}\{\text{ln}[z_{j}\left(t-\tau \right)]\}d\tau . \end{array}$$ (28)

The latter equation states that the seismic data is given by the convolution between a source wavelet and the acoustic impedance of the subsurface medium. Eq (28) can be written in a compact form as: $d^{est}=WDm$. Here, $d^{cal}\in R^{(c+n-1)\times u}$ represents the seismic data calculated by the parameters $m=\text{ln}\left(Z\right)\in R^{n\times u}$ which is used to estimate the acoustic impedance **Z**. The operators $W\in R^{\left(c+n-1)\times (n-1\right)}$ and $D\in R^{(n-1)\times n}$ are described by [76]:

$$\begin{array}{c} W=[\begin{array}{cccc} \omega_{1} & 0 & ... & 0\\ \vdots & \omega_{1} & \vdots & 0\\ \omega_{n} & \vdots & \ddots & 0\\ 0 & \omega_{n} & \vdots & \omega_{1}\\ \vdots & \vdots & \ddots & \vdots \\ 0 & ... & ... & \omega_{n} \end{array}],\;D=\frac{1}{2}[\begin{array}{ccccc} -1 & 1 & 0 & ... & 0\\ 0 & -1 & 1 & ... & 0\\ \vdots & \vdots & \ddots & \ddots & 0\\ 0 & ... & ... & -1 & 1 \end{array}]. \end{array}$$ (29)

Where W is the wavelet operator, which computes the convolution between the seismic signal and Dm. Finally, D represents the first order derivative operator. In addition, we can group these two matrices into a single operator G=WD. In this way, we compute the residuals between the observed data and the estimated data $x=d^{obs}-Gm$.

**Fig 10:**
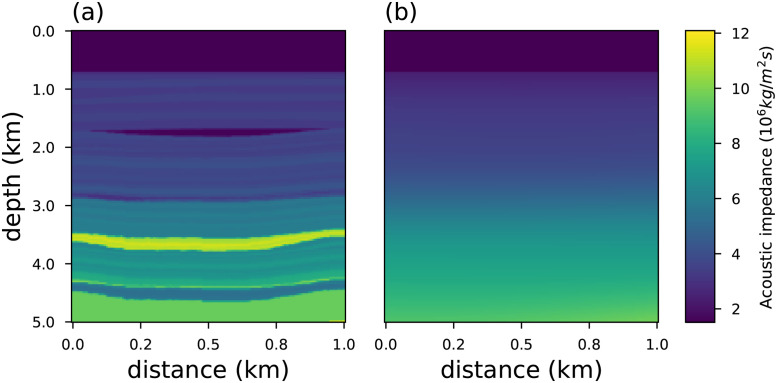
The geophysical model employed to illustrate the inversion methodology. In (a) the synthetic acoustic impedance model called Marmousi2. In (b) we show the initial model employed in the inversion methodology.

To analyze the outlier-resistance of the objective functions presented in Section 2, we consider a portion of the synthetic geological Marmousi2 [77, 78] model as a benchmark (true model). In particular, we take into account the acoustic impedance model that consists of 5*km* of depth and 1*km* of distance, as depicted in Fig 10. The seismic source used was a Ricker wavelet [79, 80] with the peak frequency *ν*_*p*_ = 55*Hz* (the most energetic frequency). For a general frequency *ν*_*p*_ the Ricker wavelet *ω*(*t*) is defined as:

$$\begin{array}{c} \omega \left(t\right)=(1-2\pi^{2}\nu_{p}^{2}t^{2})\text{exp}(-\pi^{2}\nu_{p}^{2}t^{2}). \end{array}$$ (30)

**Fig 11:**
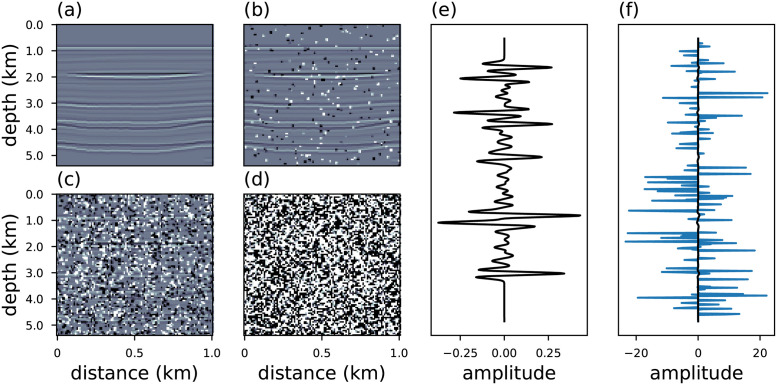
The noiseless seismic data in (a). The same data contaminated with white-noise (signal-to-noise ratio *SNR* = 80) and spike noise with (b) 0.5%, (c) 5%, and (d) 80%. The black line in panels (e) represents a single seismic trace from the middle of panel (a). The same trace contaminated by noise is represented in (f) spikes (25%).

We test the robustness of the generalized objective functions using seismic data contaminated with white Gaussian noise with low intensity (taking a signal-to-noise ratio equal to 80*dB* in all scenarios) and spikes (outliers) with different intensities, as shown in Fig 11. The spikes were added by randomly choosing positions in the seismic data and adding peaks with intensities between 5*f* and 15*f* times the original amplitude, where *f* is a Gaussian random variable with zero-mean and standard-deviation *σ*^2^ = 1. We considered 161 noise scenarios, where the difference is in the percentages of samples contaminated by the outliers. In this regard, the number of samples were chosen from 0% to 80% of the data samples, with steps of 0.5%.

For each spiky-noise scenario, we carried out data-inversions employing the *η*-, *q*- and *κ*-objective functions, Eqs (14), (20) and (26), respectively with 1/3 < *η* ≤ 1, 1 ≤ *q* < 3 and 0 ≤ *κ* ≤ 2/3 using 200 values for each of these parameters with uniform spacing between intervals. In total, for each objective function, we get 32.000 results. To minimize the objective functions, we employ the conjugate gradient method [81, 82], defining a maximum of 10 iterations and a tolerance error *ϵ* = 10^−12^.

**Fig 12:**
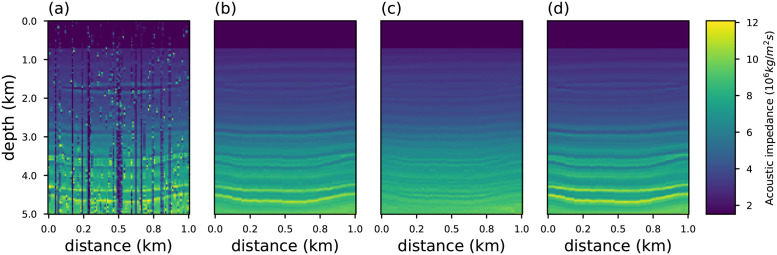
Acoustic impedance model recovered for an observed data contaminated with white noise (SNR = 80) and spike noise (0.5%) using objective function (a) conventional (b) Rényi with *η* = 0.3334; (c) Tsallis with *q* = 2.9999; and (d) Kaniadakis with *κ* = 0.6666.

**Fig 13:**
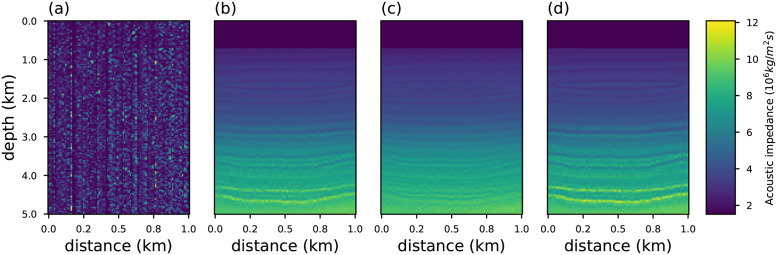
Acoustic impedance model recovered for an observed data contaminated with white noise (SNR = 80) and spike noise (80%) using objective function (a) conventional (b) Rényi with *η* = 0.3334; (c) Tsallis with *q* = 2.9999; and (d) Kaniadakis with *κ* = 0.6666.

We consider the Pearson’s correlation coefficient [83] as the statistical metric to compare the PSI results, which is defined as:

$$\begin{array}{c} R=\frac{\sum_{n=1}^{N}\Delta_{n}^{true}\Delta_{n}^{rec}}{\sqrt{\sum_{n=1}^{N}{\left(\Delta_{n}^{true}\right)}^{2}}\sqrt{\sum_{n=1}^{N}{\left(\Delta_{n}^{rec}\right)}^{2}}} \end{array}$$ (31)

where $\Delta_{n}^{true}=m_{n}^{true}-\mu^{true}$ with **m**^*true*^ = **m** and $\Delta_{n}^{est}=m_{n}^{est}-\mu^{est}$ is the difference between the true and recovered models, and their respective averages, *μ*. The coefficient R assumes values between −1 and +1. The case of R close to zero implies the absence of correlation. The correlation is strong when it approaches one. Figs 12 and 13 show the recovered acoustic impedance for conventional and generalized objective functions. We notice that the PSI results obtained with the conventional objective function are severely affected by the presence of outliers. On the other hand, at the limits *η* → 1/3, *q* → 3 and *κ* → 2/3 the influence of spikes are minimized, and good estimates are obtained.

**Fig 14:**
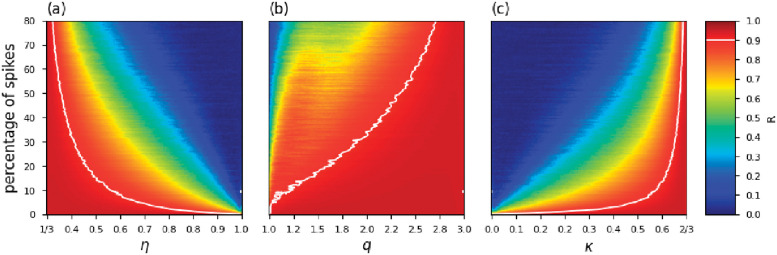
Heat-map representing the correlation between synthetic and recovered impedance models for the objective functions: (a) Rényi, (b) Tsallis and (c) Kaniadakis. The white markings indicate points such that *R* = 0.9 (strong correlation).

We summarize the PSI results for all numerical simulations performed in the present work in Fig 14. In this figure, we remark that the objective functions present satisfactory results in *η* → 1/3, *q* → 3 and *κ* → 2/3 limit cases, as predicted by the numerical experiment presented in Section 3.1. Indeed, in these limit cases, the generalized objective functions are robust tools capable of mitigating the influence of outliers, which leads to good results even for high spike contamination. In addition, it should be noted that the PSI results related with the reddish regions of the heat map in Fig 14 show a strong correlation regardless of the contamination rate employed.

We also calculated MAE and sMAPE distances between the reconstructed models in each numerical experiment and the true model. In Table 1, we present the smallest errors for each statistic for some values of the percentage of spikes (%*Sp*). Analyzing these results, we observed that the conventional approach is superior to those based on generalized statistics when the noise is purely Gaussian (%*Sp* = 0). However, when non-Gaussian errors are considered, the generalized approaches are superior to the conventional approach, especially in the Kaniadakis statistics case, which presents minor errors.

**Table 1:** Summary of smallest Mean Absolute Error (MAE) and symmetric Mean Absolute Percentage Error (sMAPE) values for objective functions based on Rényi, Tsallis and Kaniadakis frameworks. %*Sp* indicates the percentage of spikes, while *ζ* represents a normalization constant for the MAE to present this error metric best. The maximum MAE value is *ζ* = 4, 020.91, which happened in the numerical test with %*Sp* = 80 using the conventional objective function.

	Rényi			Tsallis			Kaniadakis		
%*Sp*	*η*	MAE	sMAPE	*q*	MAE	sMAPE	*κ*	MAE	sMAPE
0	1	0.073*ζ*	0.057	1	0.073*ζ*	0.057	0	0.073*ζ*	0.057
5	0.357	0.589*ζ*	0.422	2.950	0.682*ζ*	0.496	0.663	0.154*ζ*	0.114
10	0.347	0.734*ζ*	0.527	2.970	0.795*ζ*	0.575	0.663	0.201*ζ*	0.146
15	0.347	0.788*ζ*	0.570	2.940	0.840*ζ*	0.610	0.643	0.252*ζ*	0.179
20	0.350	0.837*ζ*	0.606	2.990	0.866*ζ*	0.630	0.660	0.282*ζ*	0.202
25	0.337	0.867*ζ*	0.629	2.980	0.878*ζ*	0.637	0.643	0.317*ζ*	0.225
30	0.347	0.900*ζ*	0.655	2.940	0.900*ζ*	0.662	0.647	0.364*ζ*	0.258
35	0.343	0.914*ζ*	0.666	2.980	0.907*ζ*	0.663	0.650	0.407*ζ*	0.288
40	0.350	0.930*ζ*	0.679	2.990	0.912*ζ*	0.668	0.653	0.438*ζ*	0.311
45	0.343	0.934*ζ*	0.682	2.970	0.895*ζ*	0.655	0.657	0.463*ζ*	0.325
50	0.350	0.960*ζ*	0.705	2.970	0.912*ζ*	0.666	0.660	0.517*ζ*	0.363
55	0.350	0.959*ζ*	0.700	2.960	0.934*ζ*	0.680	0.653	0.519*ζ*	0.366
60	0.337	0.977*ζ*	0.712	2.950	0.916*ζ*	0.662	0.653	0.588*ζ*	0.413
65	0.353	0.980*ζ*	0.720	2.970	0.932*ζ*	0.681	0.643	0.602*ζ*	0.428
70	0.337	0.977*ζ*	0.710	2.990	0.925*ζ*	0.677	0.643	0.637*ζ*	0.452
75	0.347	0.995*ζ*	0.728	2.950	0.942*ζ*	0.689	0.643	0.657*ζ*	0.467
80	0.340	0.991*ζ*	0.723	2.930	0.930*ζ*	0.680	0.643	0.689*ζ*	0.491

## 4 Conclusions

In this work we explore robust methods based on the Rényi, Tsallis and Kaniadakis generalized statistics. Since the solution of the data-inversion strongly depends on the employed objective function, the generalized objective functions are indeed valuable for this purpose. In fact, given a proper choice of the entropic index, it is possible to get a robust objective function that handles errors that do not obey the Gaussian statistics.

The manuscript studied three statistics: Rényi, Tsallis and Kaniadakis. Each of them is associated with a parameter *η*, *q* and *κ*. The performance of the statistics depends on the values of these parameters, as it is indicated in Fig 14. However, the parameters span along distinct ranges: 1 < *η* < 1/3, 1 < *q* < 3 and 0 < *κ* < 2/3 which make the comparison tricky. In figure (14) we equally sampled 200 points along the *x* and *y* axes in order to compare the relative performance of the statistics. In the figure is indicated by a white curve the points corresponding to correlation coefficient equal to 0.9. Considering the number of points below this curve (correlation coefficients above 0.9) a visual inspection shows that the Tsallis statistics shows a better performance in the inversion for this particular seismic problem. However, the error metrics (MAE and sMAPE) indicated that the reconstructed models using the Kaniadakis *κ*-statistics are quantitatively better and closer to the true model in the limit *κ* → 2/3.

In particular, we investigate a special example of non-Gaussian errors: outliers. In this scenario, we seek to answer some basic questions: (i) what is the most appropriate choice for entropic indexes? (ii) Which of the proposed methods is more resistant to outliers? For the first question, we find that there is a limit for every method in which the objective functions are able to ignore aberrant values without compromising the results. In addition, we note that the properties of the *η*-, *q*- and *κ*-generalized distributions and the respective objective functions have similar characteristics at the limit: (*η*, *q*, *κ*)→(1/3, 3, 2/3). These findings are relevant not only in the inverse problem theory, but also in a broad statistical context [12, 84, 85].

To conclude, it is worth emphasizing that although these methodologies have been successfully employed in geophysical applications, our proposals are easily adaptable to a wide variety of parameter estimation problems. In this regard, we hope that the methodologies proposed in this work are of great value for the modelling of complex systems with numerous unknown variables, as the generalized objective functions are able to reduce the computational cost by accelerating the convergence of the process of optimization, as shown by analyzing the influence function.
